# Classification of Lung Cancer Tumors Based on Structural and Physicochemical Properties of Proteins by Bioinformatics Models

**DOI:** 10.1371/journal.pone.0040017

**Published:** 2012-07-19

**Authors:** Faezeh Hosseinzadeh, Mansour Ebrahimi, Bahram Goliaei, Narges Shamabadi

**Affiliations:** 1 Student at Laboratory of Biophysics and Molecular Biology, Institute of Biophysics and Biochemistry, University of Tehran, Tehran, Iran; 2 Department of Biology at Basic science School & Bioinformatics Research Group, Green Research Center, University of Qom, Qom, Iran; 3 Department of Medical Physics, Iran University of Medical Science, Tehran, Iran; 4 Bioinformatics Research Group, Green Research Center, University of Qom, Qom, Iran; Howard University, United States of America

## Abstract

Rapid distinction between small cell lung cancer (SCLC) and non-small cell lung cancer (NSCLC) tumors is very important in diagnosis of this disease. Furthermore sequence-derived structural and physicochemical descriptors are very useful for machine learning prediction of protein structural and functional classes, classifying proteins and the prediction performance. Herein, in this study is the classification of lung tumors based on 1497 attributes derived from structural and physicochemical properties of protein sequences (based on genes defined by microarray analysis) investigated through a combination of attribute weighting, supervised and unsupervised clustering algorithms. Eighty percent of the weighting methods selected features such as autocorrelation, dipeptide composition and distribution of hydrophobicity as the most important protein attributes in classification of SCLC, NSCLC and COMMON classes of lung tumors. The same results were observed by most tree induction algorithms while descriptors of hydrophobicity distribution were high in protein sequences COMMON in both groups and distribution of charge in these proteins was very low; showing COMMON proteins were very hydrophobic. Furthermore, compositions of polar dipeptide in SCLC proteins were higher than NSCLC proteins. Some clustering models (alone or in combination with attribute weighting algorithms) were able to nearly classify SCLC and NSCLC proteins. Random Forest tree induction algorithm, calculated on leaves one-out and 10-fold cross validation) shows more than 86% accuracy in clustering and predicting three different lung cancer tumors. Here for the first time the application of data mining tools to effectively classify three classes of lung cancer tumors regarding the importance of dipeptide composition, autocorrelation and distribution descriptor has been reported.

## Introduction

Lung cancer is a leading cause of deaths from cancer worldwide. Among lung cancers, non-small cell lung cancer (NSCLC) affects about 80% of patients and, when diagnosed at a localized stage, the 5-year survival is about 50%, whereas it decreases to 8% and 3% in the case of lymph node involvement or metastasis, respectively [1]. Inhalation of tobacco smoke and other environmental carcinogens is considered a major etiologic factor [2]. Epidemiologic studies continue to provide evidence that genetic variability in the individual response to carcinogens might modify the susceptibility to cancer. Polymorphisms of genes involved in detoxification of carcinogens, and those that modulate and repair DNA damage after carcinogen exposure, have been linked to the risks of lung cancer [3].

Patients with non-small cell lung tumors (squamous, AC, and large cell) are treated differently from those with small cell tumors, therefore pathological distinction between these two types of lung tumor is very important. The gene expression patterns made possible the sub classification of adenocarcinoma into subgroups that correlated with the degree of tumor differentiation as well as patient survival. Gene expression analysis thus promises to extend and refine standard pathologic analysis [4]. It has been widely accepted that lung carcinogenesis is a multistep process and phenotypic changes resulted from activation of oncogenes and inactivation of tumor suppressor genes [5]. Non-small cell lung cancer (NSCLC) is the leading cause of cancer mortality worldwide. At present no reliable biomarkers are available to guide the management of this condition. Microarray technology may allow appropriate biomarkers to be identified but present platforms are lacking disease focus and are thus likely to miss potentially vital information contained in patient tissue samples. A combination of large-scale in-house sequencing, gene expression profiling and public sequence and gene expression data mining were used to characterize the transcriptome of NSCLC [6]. Identifying a useful prognostic biologic and molecular marker is therefore important to evaluate the biologic and molecular characteristics that differed from tumor, lymph node, metastasis TNM staging in non-small cell lung cancer (NSCLC) in order to predict prognosis and establish preventive methods [7]. A better understanding of the molecular pathogenesis of SCLC would likely suggest strategies for earlier diagnosis and new molecular-targeted therapies [8].

In recent studies, some classifiers are used for classification of cancer genes or proteins, for example KNN classifier can have some utility for some microarray classification problems, acting on the entire non-dimension reduced dataset. They show that increasing the dimensionality of these sets (considering pairs, triples or four-tuples, rather than individual transcript sequences one by one) can lead to significant improvements with each dimension gained [9]. In other study, features of proteins expressed in malignant, benign and both cancers were compared using different screening techniques, clustering methods, decision tree models and generalized rule induction (GRI) algorithms to look for patterns of similarity in two benign and malignant breast cancer groups [10] or developing and testing a naive Bayesian classifier based on sequence properties of the genes and the molecular function and biological processes in which they are involved in order to unveil their unique features that can assist towards the identification of new candidate cancer genes [11] or implementing a systematic method that predicts cancer involvement of genes by integrating heterogeneous datasets by relying on: (i) protein-protein interactions; (ii) differential expression data; and (iii) structural and functional properties of cancer genes [12].

Also in the classification of lung cancer, in several studies, the data mining models have been used. For example a classification and regression tree (CART) model was trained to classify 41 clinical specimens as disease/nondisease based on 26 variables computed from the mass-to-charge ratio (m/z) and peak heights of proteins identified by mass spectroscopy of blood serum samples from people with and without lung cancer [13], or a training-testing approach to the molecular classification of resected non-small cell lung cancer that in this study, a training-testing approach has been used to test the reliability of cDNA microarray-based classifications of resected human non-small cell lung cancers (NSCLCs) analyzed by cDNA microarray [14]. In the other study, classification of individual lung cancer cell lines (SCLC and NSCLC) has been performed based on DNA methylation markers by using of linear discriminant analysis and artificial neural networks, and in the result, this work supports the promise of ANN analysis of DNA methylation data as a powerful approach for the development of automated methods for lung cancer classification [15]. In another study lung cancer gene expression database analysis incorporated prior knowledge with support vector machine-based classification method, together with the application of support vector machine as the discriminant approach, and a method proposed that incorporated prior knowledge into cancer classification based on gene expression data to improve accuracy [16]. To automatically classify lung tumor-node-metastases (TNM) cancer stages from free-text pathology reports using symbolic rule-based classification. The accuracy measure and confusion matrices were used to evaluate the TNM stages classified by the symbolic rule-based system. The system was evaluated against a database of multidisciplinary team staging by decisions and a machine learning-based text classification system using support vector machines [17]. Sequence-derived structural and physicochemical features have frequently been used in the development of statistical learning models for predicting proteins and peptides of different structural, functional and interaction profiles.

PROFEAT (Protein Features) is a web server for computing COMMONly-used structural and physicochemical features of proteins and peptides from amino acid sequence [18]. Sequence-derived structural and physicochemical features have frequently been used for predicting protein structural and functional classes [19], [20], [21], [22], [23], protein–protein interactions [24], [25], [26], subcellular locations [27], [28] and peptides of specific properties [29] from their sequence. These features are highly useful for representing and distinguishing proteins or peptides of different structural, functional and interaction profiles, which is essential for the successful application of statistical learning methods in predicting the structural, functional and interaction profiles of proteins and peptides irrespective of sequence similarity [30].

In this study, with attention to the importance of classification of lung tumors in diagnosis and treatment of this disease and application and usefulness of sequence-derived structural and physicochemical features of proteins, classification of 2 types of lung tumors based on the structural and physicochemical properties of proteins investigated by using of bioinformatics and data mining tools.

## Materials and Methods

### Data Preparation

Microarray analysis on GSEA db (Gene Set Enrichment Analysis database) used to extract genes involved in either type of lung tumors (SCLC or NSCLC). Some genes were COMMON in both tumors so named as COMMON set. Proteins for each group of genes (SCLC = 59, NSCLC = 30 or COMMON = 25) extracted by DAVID server (http://david.abcc.ncifcrf.gov) and protein sequences extracted from UniProt Knowledgebase (Swiss-Prot and TrEmble) database. One thousands and ninety seven protein features or attributes computed by PROFEAT web (http://jing.cz3.nus.edu.sg/cgi-bin/prof/prof.cgi) including structural and physicochemical protein. An index Fi.j.k.l is used to represent the l^th^ descriptor value of the k^th^ descriptor of the j^th^ feature in the i^th^ feature group, which serves as an easy reference to the PROFEAT manual provided in the server homepage and a lists of these feature groups showed in Table S1 (details have presented in Appendix S1) [18]. A dataset of these protein features was imported into Rapid Miner (Rapid Miner 5.0.001, Rapid-I GmbH, Stochumer Str. 475, 44227 Dortmund, Germany) software, and the type of tumor (SCLC, NSCLC or COMMON) was set as the target or label attribute.

### Data Cleaning

Duplicate features removed by comparing all examples with each other on the basis of the specified selection of attributes (two examples were assumed equal if all values of all selected attributes were equal). Then useless attributes removed from the dataset. Numerical attributes which possessed standard deviations less than or equal to a given deviation threshold (0.1) assumed as to be useless and removed. Finally, correlated features (with Pearson correlation greater than 0.9) omitted. After cleaning, the number of attributes and records decreased and this database labeled as Final Cleaned database (FCdb).

### Attribute Weighting

To identify the most important features and to find the possible patterns in features that contribute to lung cancer tumors, 10 different algorithms of attribute weightings were applied to the cleaned dataset (FCdb) as described below.

#### Weight by information gain

*This operator calculated the relevance of a feature by computing the information gain in class distribution.*

#### Weight by information gain ratio

*This operator calculated the relevance of a feature by computing the information gain ratio for the class distribution.*

#### Weight by rule

*This operator calculated the relevance of a feature by computing the error rate of a OneR Model on the example set without this feature.*

#### Weight deviation

*This operator created weights from the standard deviations of all attributes. The values were normalized by the average, the minimum, or the maximum of the attribute.*

#### Weight by chi squared statistic

*This operator calculated the relevance of a feature by computing, for each attribute of the input example set, the value of the chi-squared statistic with respect to the class attribute.*

#### Weight by Gini index

*This operator calculated the relevance of an attribute by computing the Gini index of the class distribution, if the given example set would have been split according to the feature.*

#### Weight by uncertainty

*This operator calculated the relevance of an attribute by measuring the symmetrical uncertainty with respect to the class.*

#### Weight by relief

*This operator measured the relevance of features by sampling examples and comparing the value of the current feature for the nearest example of the same and of a different class. This version also worked for multiple classes and regression data sets. The resulting weights were normalized into the interval between 0 and 1.*

#### Weight by SVM (Support Vector Machine)

*This operator used the coefficients of the normal vector of a linear SVM as feature weights.*

#### Weight by PCA (Principle Component Analysis)

*This operator used the factors of the first of the principal components as feature weights.*

### Attribute Selection

After attribute weighting models ran on the FCdb, each protein attribute (feature) gained a value between 0 and 1, which revealed the importance of that attribute with regards to a target attribute (type of tumors). All variables with weights higher than 0.50 were selected and 10 new datasets created. These newly formed datasets were named according to their attribute weighting models (Information gain, Information gain ratio, Rule, Deviation, Chi Squared, Gini index, Uncertainty, Relief, SVM and PCA) and were used to join with subsequent models (supervised and unsupervised). Each model of supervised or unsupervised clustering were performed 11 times; the first time it was run on the main dataset (FCdb) and then on the 10 newly formed datasets (the results of attribute weighting).

### Unsupervised Clustering Algorithms

The clustering algorithms listed below were applied on the 10 newly created datasets (generated as the outcomes of 10 different attribute weighting algorithms (as well as the main dataset (FCdb).

#### K-Means

This operator uses kernels to estimate the distance between objects and clusters. Because of the nature of kernels, it is necessary to sum over all elements of a cluster to calculate one distance.

#### K-Medoids

This operator represents an implementation of k-Medoids. This operator will create a cluster attribute if it is not yet present.

### Tree Induction Models

#### DecisionTrees

Five tree induction models including Decision Tree, Decision Tree Parallel, Decision Stump, Random Tree and Random Forest ran on the main dataset (FCdb). A weight-based parallel decision tree model, which learns a pruned decision tree based on an arbitrary feature relevance test (attribute weighting scheme as inner operator), applied to 10 different datasets created from attribute weighting selection (SVM, Gini Index, Uncertainty, PCA, Chi Squared, Rule, Relief, Information Gain, Information Gain Ratio and Deviation).

### Machine Based Prediction by Leave One-out 10-fold Cross Validation

#### Decision Tree

Sixteen machine learning models run on four decision tree algorithms (*Decision Tree, Decision Tree Parallel, Decision Stump* and *Random Forest*) with four different criteria (*Gain Ratio, Information Gain, Gini Index* and *Accuracy*) on all 11 datasets to find a suitable model(s) to predict the accuracies and the classification errors of classes based on protein attributes. To calculate the accuracy of each model, 10-fold cross validation [14] is used to train and test models on all patterns. To perform cross validation, all the records were randomly divided into 10 parts, 9 sets were used for training and the 10th one for testing (leave one-out). The process was repeated 10 times and the accuracy for true, false and total accuracy calculated. The final accuracy reported as the average of the accuracy in all ten tests.

## Results

### Data Cleaning

The initial dataset contained 114 records (protein sequences) with 1497 protein features. Of these records, 59 records were classified as SCLC class, 30 records belonged to NSCLC class and 25 records were classified as COMMON class. Following removal of duplicates, useless attributes, and correlated features (data cleaning) the number of protein features decreased to 1089 features.

### Attribute Weighting

Data were normalized before running the models; it was expected that all weights would be between 0 and 1. Features gained weight values higher than 0.50 with at least 50% of weighting algorithms regarded as important protein features (Table S2).

### Unsupervised Clustering Algorithms

Two different unsupervised clustering algorithms (K-Means and K-Medoids) were applied on FCdb and ten datasets created using attribute selection (weighting) algorithms. None of clustering algorithms were able to differentiate fully the proteins that involved in any types of lung tumor (Table S3).

### Tree Induction Models

Five tree induction models (Decision Tree, Decision Tree Parallel, Decision Stump, Random Tree and Random Forest) ran on FCdb and 10 datasets that generated after performing 10 attribute weighting algorithms. In total 151 trees generated (Random Forest model itself included 10 models).

Several models induced simple trees while others were complicated; 9 Decision Tree and 35 Random Forest models were the best trees to clearly distinguish between two cancer types.

**Figure 1:**
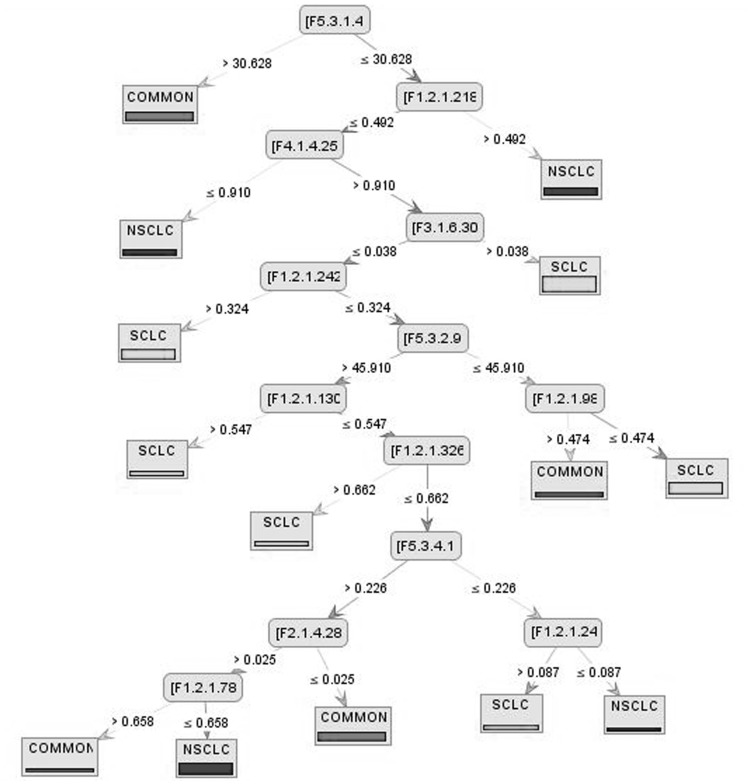
Decision Tree model on Information Gain dataset.

Distribution of hydrophobicity was the most important attribute used to build the tree when the Decision Tree model applied to Information Gain dataset (Figure 1). When the value for this feature was more than 30.628, the proteins fell into the COMMON class. The autocorrelation descriptors and dipeptide compositions were the other features used to build the rest of the tree. If composition of Cysteine-Glutamic acid ([F1.2.1.24]: polar dipeptide) was more than 0.087, the protein belonged to SCLC tumor and otherwise fell into NSCLC class. Composition of nonpolar dipeptides in NSCLC proteins was more than SCLC proteins ([F1.2.1.218]: Met-Val) and overhand, dipeptide compositions of SCLC proteins are more polar than NSCLC proteins ([F1.2.1.326]: Thr-Gly, [F1.2.1.98]: Phe-Val). The details of this model have become at the below.

**Figure 2:**
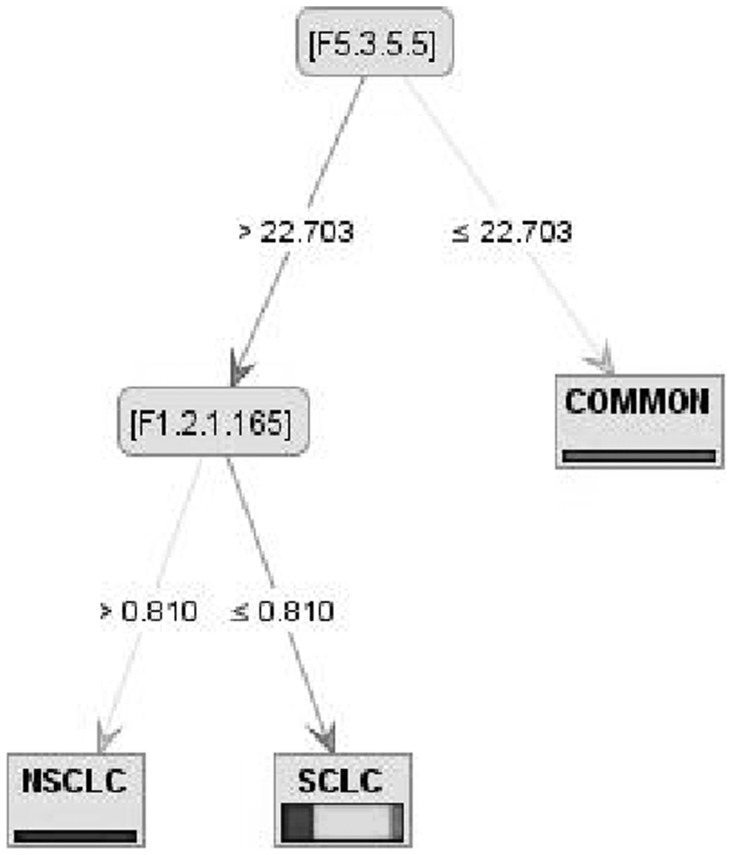
Model 1 of Random Forest on SVM dataset. In the first step if distribution of charge was equal to or lower than 22.703 the proteins fell into COMMON class; dipeptide composition was other important feature for drawing this tree.

Following important points can be extracted from the tress in general, these results have reported for the first time:

1. F1.2 (dipeptide composition), F3.1 (Moran autocorrelation) and F5.3 (distribution descriptor) were the most important protein features used by decision tree models to classify three lung cancer classes (SCLC, NSCLC, COMMON).
2. Distribution of hydrophobicity (F5.3.1) in COMMON class was very high while the distribution of charges (F5.3.5) was very low (Figure 2).
3. Generally the composition of non-polar dipeptides in SCLC class was smaller than COMMON proteins and composition of polar dipeptide in SCLC associated proteins was higher than NSCLC class (Figure 1).

### Machine Based Prediction by Leave One-out 10-fold Cross Validation

The accuracies of all induced prediction algorithms are presented in Table S4. Nearly, the average accuracies of all models showed accuracies higher than 60%. The lowest accuracies gained when Stump Decision Tree model ran on Relief dataset with Gini Index criteria (41.89%). The best predicted accuracy achieved when Random Forest Decision Tree model ran on Rule dataset with Gain Ratio (86.00%).

## Discussion

Lung cancer can be roughly divided into two groups according to pathology: non-small cell lung cancer (NSCLC) (80.4%) and small cell lung cancer (16.8%) [31]. Patients with non-small cell lung tumor are treated differently from those with small cell tumors. The pathological distinction between small cell lung cancer (SCLC) and non-small cell lung cancer is, therefore, very important [4]. Many studies have considered to classification of lung cancer [16], [32], [33], [34], [35], [36], [37], [38], [39]. For example, RNA expression patterns associated with non-small cell lung cancer sub classification have been reported, but there are substantial differences in the key genes and clinical features of these subsets casting doubt on their biological significance. In this recent study, a training-testing approach have used to test the reliability of cDNA microarray-based classifications of resected human non-small cell lung cancers (NSCLCs) analyzed by cDNA microarray. These results demonstrated that gene expression profiling can identify molecular classes of resected NSCLCs that correctly classifies a blinded test cohort, and correlates with and supplements standard histological evaluation [14]. In summary, extensive and detailed support for the idea that gene expression-based classification of tumors will soon become clinically useful for cancer of the lung have provided [4]. Molecular classification of NSCLC using an objective quantitative test can be highly accurate and could be translated into a diagnostic platform for broad clinical application [40].

Sequence-derived structural and physicochemical descriptors have frequently been used in machine learning prediction of protein structural and functional classes [19], [20], [21], [22], [23], [24], protein-protein interactions [24], [25], [26], [41], subcellular locations [27], [28], [42], [43], peptides containing specific properties [29], [44], microarray data [45] and protein secondary structure prediction [46]. These descriptors serve to represent and distinguish proteins or peptides of different structural, functional and interaction profiles by exploring their distinguished features in compositions, correlations, and distributions of the constituent amino acids and their structural and physicochemical properties [18], [20], [26], [30] and this proved that currently used descriptor-sets are generally useful for classifying proteins and the prediction performance may be enhanced by exploring combinations of descriptors [47].

In present study, we used structural and physicochemical properties of proteins that involve in any types of lung tumors for classification of them and detecting most important protein properties that have participated in distinguish of lung tumors. Various modeling techniques were applied to study 1497 attributes of proteins that involved in two and four types (unpublished data) of lung cancer. When the number of variables or attributes is sufficiently large, the ability to process units is significantly reduced. Data cleansing algorithms were used to remove correlated, useless or duplicated attributes which results in a smaller database [48], [49]. About 15% of the attributes discarded when these algorithms were applied on the original datasets.

Ten different attribute weighting models applied on final cleaned dataset; as each algorithm uses a specific pattern to define the most important features, thus, the results may be different [50]. The feature groups of F5.3 (distribution descriptors), F1.2 (dipeptide composition) and F3.1 (autocorrelation ) were the most important attributes selected by attribute weighting models to distinguish between SCLC, NSCLC and COMMON classes of lung tumor types, as defined by 80% of the attribute weighting algorithms (Table S2).

Furthermore in the appropriate decision trees, compatible results with attribute weighting algorithms were shown and the same protein attribute groups (F2.1, F3.1, F5.3 and F1.2) selected as the most important attributes in classification of lung tumor proteins. In addition, most induced trees showed F5.3 attributes, distribution of hydrophobicity in COMMON proteins was very high and distribution of charged residues in these proteins was very low, therefore the results confirmed proteins from COMMON class were very hydrophobic.

The importance of hydrophobicity has been highlighted in some studies [51], [52], [53]. It is well known that hydrophobicity plays a major role in determining the properties of amino acids, peptides and proteins. In another study, hydrophobic residues were predominant in slow range of folding, and hydrophilic residues frequently occurred in fast range. In general, the surrounding environment of proteins is water. Typically, the side-chains of hydrophobic residues are buried in the interior of proteins to form hydrophobic core, which is apart from water, while the side-chains of hydrophilic residues are exposed to the surface of proteins, which is close to water molecular [54]. Therefore, the results of our study, for the first time, confirm that the importance of hydrophobicity in allowing fast folding of the COMMON proteins between two types of lung tumors and increasing their capability for tumorigenic property.

Dipeptide composition was other important protein feature groups selected as an important in present study. In our recent studies, we showed that specific dipeptides play the central role in classification of breast cancer and protein halo stability and thermo stability [10], [55], [56]. The importance of sequence-based classification in detection of various proteins expressed in breast cancer and the importance of Ile-Ile dipeptide in clustering of proteins, were reported there [10]. In this paper, most of decision tree models showed that composition of polar dipeptide in SCLC proteins were more than NSCLC proteins and vice versa, resulting NSCLC proteins to show more hydrophobicity. These results have reported for the first time and may be one of the main factors to facilitate SCLC tumors distribution.

In present study, autocorrelation descriptor was another important feature group for classification of lung tumors. Autocorrelation descriptors are a class of topological descriptors, also known as molecular connectivity indices, describe the level of correlation between two objects (protein or peptide sequences) in terms of their specific structural or physicochemical property [57], which are defined based on the distribution of amino acid properties along the sequence [58]. Eight amino acid properties are used for deriving the autocorrelation descriptors: hydrophobicity scale [59]; average flexibility index [60]; polarizability parameter [61]; free energy of amino acid solution in water [61]; residue accessible surface areas [62]; amino acid residue volumes [63]; steric parameters [64]; and relative mutability [65]. One of recent study proved that the AASA (amino acid sequence autocorrelation) information is very effective to represent the relationship between the protein sequence and corresponding folding rates [54]. So the autocorrelation properties may play an important role in folding of three lung cancer tumors studied here and this feature has been reported for the first time in this study. Autocorrelation approach had successful usage for modeling molecular properties, biological activities [66], [67] and prediction of protein helix content [68]. In a recent study, a method for reconstructing the strain distribution by modifying the autocorrelation technique, “combined autocorrelation method” proposed. In the experiments using a tumor phantom and an extracted breast tissue including a cancer tumor, each elastic modulus image obtained by the combined autocorrelation method and the 3-D finite element tissue model clearly displayed the region harder than surrounding soft material or tissue. These results reveal that the combined autocorrelation method is a promising means for diagnosing tumors [69] as shown in this paper.

Unsupervised clustering algorithms have been widely employed in a variety of areas in the biological sciences, including diagnostics and image processing [70], EST [71], cancer detection [72], promoter analysis [71], gene and protein bioinformatics [56], [73], [74], [75], [76]. Here, we used two different unsupervised clustering methods (K-Means and K-Medoids) on FCdb and 10 datasets created from protein attributes, which were assigned high weights. The performances of these algorithms varied significantly. Some methods were able to nearly assign NSCLC protein into the correct class (for example, the K-Medoids algorithm, when applied to FCdb and Deviation, Gini Index, Information Gain, PCA and Uncertainty datasets). The results showed that the K-Medoids algorithm was nearly able to classify SCLC proteins into the correct class when runs on the Chi Squared dataset. But none of clustering algorithm was able to correctly classify COMMON proteins into respective class (Table S3). For more exact clustering of proteins that belonged to any types of lung tumors, other clustering models such as EM applied to data with higher accuracies (unpublished data).

As shown in Table S4, the overall accuracies for tree induction models were generally high enough and improved when the criteria changed. For example, the accuracy for Decision Tree Stump model for Accuracy criterion was 41.89%, but improved to 84.00% when the criterion changed indicating a very sharp increase in the model accuracy and performance. The best accuracy achieved when the Random Forest model ran with Gain Ratio criterion (86.00%) which makes it the best model to apply in such conditions and is the first machine based learning algorithm to predict lung cancer tumor types based on protein attributes.

To our best knowledge, the findings of this study for the first time showed that protein features can be effectively used to determine any types of lung cancer tumors. Dipeptide composition, Moran autocorrelation and distribution descriptor were the most important protein features selected by bioinformatics tools. Also for the first time, we showed SCLC proteins were more hydrophilic than NSCLC.

## Supporting Information

Table S1 **The indices of protein feature groups computed by PROFEAT web server for each protein sequence.**
(DOCX)

Table S2 **The most important protein attributes selected by at least 5 attribute weighting algorithms.**
(DOCX)

Table S3 **Clustering of 11 datasets (FCdb and 10 datasets that generated after performing 10 attribute weighting algorithms) into SCLC, NSCLC and COMMON classes by two different unsupervised clustering algorithms (K-Means and K-Medoids).**
(DOCX)

Table S4 **The accuracy of four different tree induction models (each ran with four criteria, Accuracy, Gain Ratio, Gini Index and Info Gain) on 11 datasets computed by 10-fold cross validation.**
(DOCX)

Appendix S1 **The table shows the complete descriptions for protein attributes computed and used in this study.**
(DOCX)
