# Supplementary material for: Classification of Lung Cancer Tumors Based on Structural and Physicochemical Properties of Proteins by Bioinformatics Models
Source: PLoS One. 2012 Jul 19;7(7):e40017. doi: 10.1371/journal.pone.0040017 (PMC3400626; doi:10.1371/journal.pone.0040017)
Supplement: Table S1 — The indices of protein feature groups computed by PROFEAT web server for each protein sequence. (DOCX) [file pone.0040017.s001.docx]

Table S1. The indices of protein feature groups computed by PROFEAT web server for each protein. sequence.

| [F1.1] Aminoacid composition (%) |
| --- |
| [F1.2] Dipeptide composition (%) |
| [F2.1] Normalized Moreau-Broto autocorrelation |
| [F3.1] Moran autocorrelation |
| [F4.1] Geary autocorrelation |
| [F5.1] Composition |
| [F5.2] Transition |
| [F5.3] Distribution |
| [F6.1] Sequence-order-coupling number |
| [F6.2] Quasi-sequence-order descriptors |
| [F7.1] Pseudo aminoacid composition |
