# Supplementary material for: Classification of Lung Cancer Tumors Based on Structural and Physicochemical Properties of Proteins by Bioinformatics Models
Source: PLoS One. 2012 Jul 19;7(7):e40017. doi: 10.1371/journal.pone.0040017 (PMC3400626; doi:10.1371/journal.pone.0040017)
Supplement: Table S2 — The most important protein attributes selected by at least 5 attribute weighting algorithms. (DOCX) [file pone.0040017.s002.docx]

Table S2. The most important protein attributes selected by at least 5 attribute weighting algorithms.

| Attributes | Percent | | Attributes | Percent | Attributes | Percent | Attributes | Percent |
| --- | --- | --- | --- | --- | --- | --- | --- | --- |
| [F5.3.1.4] | 8 | [F5.3.1.13] | | 6 | [F5.3.2.5] | 5 | [F1.2.1.130] | 5 |
| [F1.2.1.244] | 8 | [F5.3.3.5] | | 6 | [F1.2.1.212] | 5 | [F1.2.1.71] | 5 |
| [F3.1.1.16] | 8 | [F2.1.3.7] | | 6 | [F1.2.1.242] | 5 | [F3.1.1.22] | 5 |
| [F3.1.4.4] | 8 | [F6.2.2.42] | | 6 | [F3.1.4.14] | 5 | [F3.1.8.23] | 5 |
| [F1.2.1.144] | 7 | [F3.1.5.23] | | 6 | [F7.1.1.38] | 5 | [F2.1.1.4] | 5 |
| [F5.3.2.6] | 7 | [F3.1.3.2] | | 6 | [F3.1.6.30] | 5 | [F5.3.6.6] | 5 |
| [F3.1.4.1] | 7 | [F3.1.2.29] | | 6 | [F1.2.1.273] | 5 | [F5.3.5.4] | 5 |
| [F1.2.1.309] | 6 | [F4.1.4.28] | | 6 | [F3.1.1.18] | 5 | [F3.1.8.18] | 5 |
| [F6.1.1.1] | 6 | [F3.1.4.28] | | 6 | [F1.2.1.149] | 5 | [F3.1.4.2] | 5 |
| [F5.3.7.4] | 6 | [F1.2.1.100] | | 5 | [F6.2.1.15] | 5 | [F4.1.6.13] | 5 |
| [F1.2.1.218] | 6 | [F1.2.1.120] | | 5 | [F3.1.3.13] | 5 | [F3.1.4.18] | 5 |
