# Supplementary material for: Classification of Lung Cancer Tumors Based on Structural and Physicochemical Properties of Proteins by Bioinformatics Models
Source: PLoS One. 2012 Jul 19;7(7):e40017. doi: 10.1371/journal.pone.0040017 (PMC3400626; doi:10.1371/journal.pone.0040017)
Supplement: Table S3 — Clustering of 11 datasets (FCdb and 10 datasets that generated after performing 10 attribute weighting algorithms) into SCLC, NSCLC and COMMON classes by two different unsupervised clustering algorithms (K-Means and K-Medoids). (DOCX) [file pone.0040017.s003.docx]

Table S3. Clustering of 11 datasets (FCdb and 10 datasets that generated after performing 10 attribute weighting algorithms) into SCLC, NSCLC and COMMON classes by two different unsupervised clustering algorithms (K-Means and K-Medoids).

| **Dataset** | **Tumor Types** | **K-Means** | **K-Medoids** |
| --- | --- | --- | --- |
|  | SCLC (59) | 42 | 75 |
| **FC data** | NSCLC (30) | 38 | **35** |
|  | COM (25) | 34 | 4 |
|  | SCLC (59) | 42 | **57** |
| **Chi Squared** | NSCLC (30) | 38 | 48 |
|  | COM (25) | 34 | 9 |
|  | SCLC (59) | 43 | 75 |
| **Deviation** | NSCLC (30) | 38 | **35** |
|  | COM (25) | 33 | 4 |
|  | SCLC (59) | 42 | 75 |
| **Gini Index** | NSCLC (30) | 38 | **35** |
|  | COM (25) | 34 | 4 |
|  | SCLC (59) | 42 | 75 |
| **Information Gain** | NSCLC (30) | 38 | **35** |
|  | COM (25) | 34 | 4 |
|  | SCLC (59) | 42 | 101 |
| **Information Gain Ratio** | NSCLC (30) | 38 | 12 |
|  | COM (25) | 34 | 1 |
|  | SCLC (59) | 43 | 75 |
| **PCA** | NSCLC (30) | 38 | **35** |
|  | COM (25) | 33 | 4 |
|  | SCLC (59) | 42 | 79 |
| **Relief** | NSCLC (30) | 38 | 19 |
|  | COM (25) | 34 | 16 |
|  | SCLC (59) | 42 | 107 |
| **Rule** | NSCLC (30) | 38 | 4 |
|  | COM (25) | 34 | 3 |
|  | SCLC (59) | 42 | 102 |
| **SVM** | NSCLC (30) | 38 | 11 |
|  | COM (25) | 34 | 1 |
|  | SCLC (59) | 42 | 75 |
| **Uncertainty** | NSCLC (30) | 38 | **35** |
|  | COM (25) | 34 | 4 |
