# Supplementary material for: Classification of Lung Cancer Tumors Based on Structural and Physicochemical Properties of Proteins by Bioinformatics Models
Source: PLoS One. 2012 Jul 19;7(7):e40017. doi: 10.1371/journal.pone.0040017 (PMC3400626; doi:10.1371/journal.pone.0040017)
Supplement: Table S4 — The accuracy of four different tree induction models (each ran with four criteria, Accuracy, Gain Ratio, Gini Index and Info Gain) on 11 datasets computed by 10-fold cross validation. (DOCX) [file pone.0040017.s004.docx]

Table S4. The accuracy of four different tree induction models (each ran with four criteria, Accuracy, Gain Ratio, Gini Index and Info Gain) on 11 datasets computed by 10-fold cross validation.

|  | **Decision Tree** | | | | **Decision Tree Parallel** | | | | **Decision Tree Stump** | | | | **Decision Tree Random Forest** | | | |  |
| --- | --- | --- | --- | --- | --- | --- | --- | --- | --- | --- | --- | --- | --- | --- | --- | --- | --- |
|  | ***Accuracy*** | ***Gain Ratio*** | ***Gini Index*** | ***Info Gain*** | ***Accuracy*** | ***Gain Ratio*** | ***Gini Index*** | ***Info Gain*** | ***Accuracy*** | ***Gain Ratio*** | ***Gini Index*** | ***Info Gain*** | ***Accuracy*** | ***Gain Ratio*** | ***Gini Index*** | ***Info Gain*** |  |
| **Chi Squared** | 62.27% | 62.27% | 62.27% | 62.27% | 58.86% | 55.98% | 58.71% | 59.55% | 58.79% | 59.62% | 58.71% | 58.71% | 55.30% | 52.58% | 60.45% | 61.21% |  |
| **Info Gain** | 51.67% | 51.67% | 51.67% | 51.67% | 51.67% | 51.67% | 53.71% | 51.67% | 51.67% | 51.67% | 51.67% | 51.67% | 51.67% | 51.67% | 54.62% | 51.67% |  |
| **Deviation** | 63.86% | 63.86% | 63.86% | 63.86% | 62.05% | 58.48% | 49.32% | 50.98% | 56.06% | 59.62% | 44.70% | 50.53% | 52.50% | 51.67% | 49.24% | 54.39% |  |
| **Gini Index** | 61.52% | 61.52% | 61.52% | 61.52% | 64.32% | 53.56% | 56.97% | 50.83% | 58.79% | 59.62% | 44.70% | 50.53% | 50.76% | 53.41% | 52.73% | 61.29% |  |
| **Info Gain Ratio** | 60.53% | 60.53% | 60.53% | 60.53% | 64.24% | 58.71% | 49.92% | 55.53% | 58.79% | 59.62% | 44.70% | 50.53% | 53.48% | 52.58% | 66.97% | 66.74% |  |
| **PCA** | 64.70% | 64.70% | 64.70% | 64.70% | 69.92% | 65.61% | 49.17% | 49.92% | 58.79% | 59.62% | 50.15% | 59.62% | 52.58% | 51.67% | 55.08% | 56.89% |  |
| **Relief** | 51.67% | 51.67% | 51.67% | 51.67% | 51.67% | 51.67% | 53.71% | 51.67% | 51.67% | 51.67% | 51.67% | 51.67% | 51.67% | 51.67% | 54.62% | 51.67% |  |
| **Rule** | 56.14% | 56.14% | 56.14% | 56.14% | 58.94% | 62.20% | 46.21% | 48.94% | 58.79% | 59.62% | **41.89%** | 48.71% | 52.50% | 50.68% | 66.52% | 57.73% |  |
| **Uncertainty** | 77.00% | 77.00% | 77.00% | 77.00% | 76.50% | 79.00% | 72.00% | 74.50% | 79.00% | 84.00% | 84.00% | 84.00% | 81.50% | **86.00%** | 81.50% | 85.50% |  |
| **SVM** | 58.03% | 58.03% | 58.03% | 58.03% | 59.62% | 57.05% | 62.12% | 58.79% | 59.62% | 59.62% | 45.61% | 56.89% | 51.67% | 54.24% | 55.98% | 55.23% |  |
| **FCdb** | 56.89% | 56.89% | 56.89% | 56.89% | 61.36% | 61.06% | 59.02% | 48.18% | 58.79% | 59.62% | 42.88% | 52.35% | 51.67% | 52.58% | 66.67% | 59.39% |  |

This table presents the accuracy percentage of Tree Induction models (*Decision Tree, Decision Tree Parallel, Decision Stump, Random Forest and Random Tree*) run with four different criteria (*Gain Ratio, Information Gain, Gini Index and Accuracy*). The lowest and highest accuracies have been underlined.
